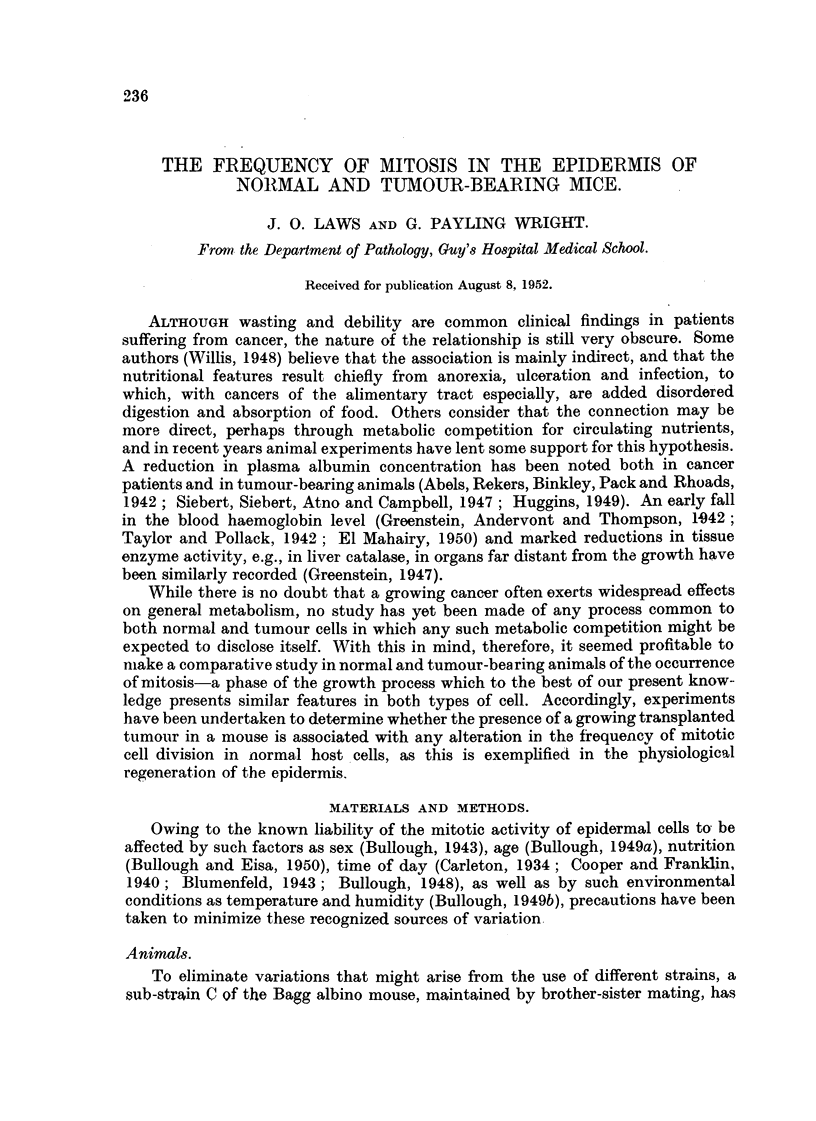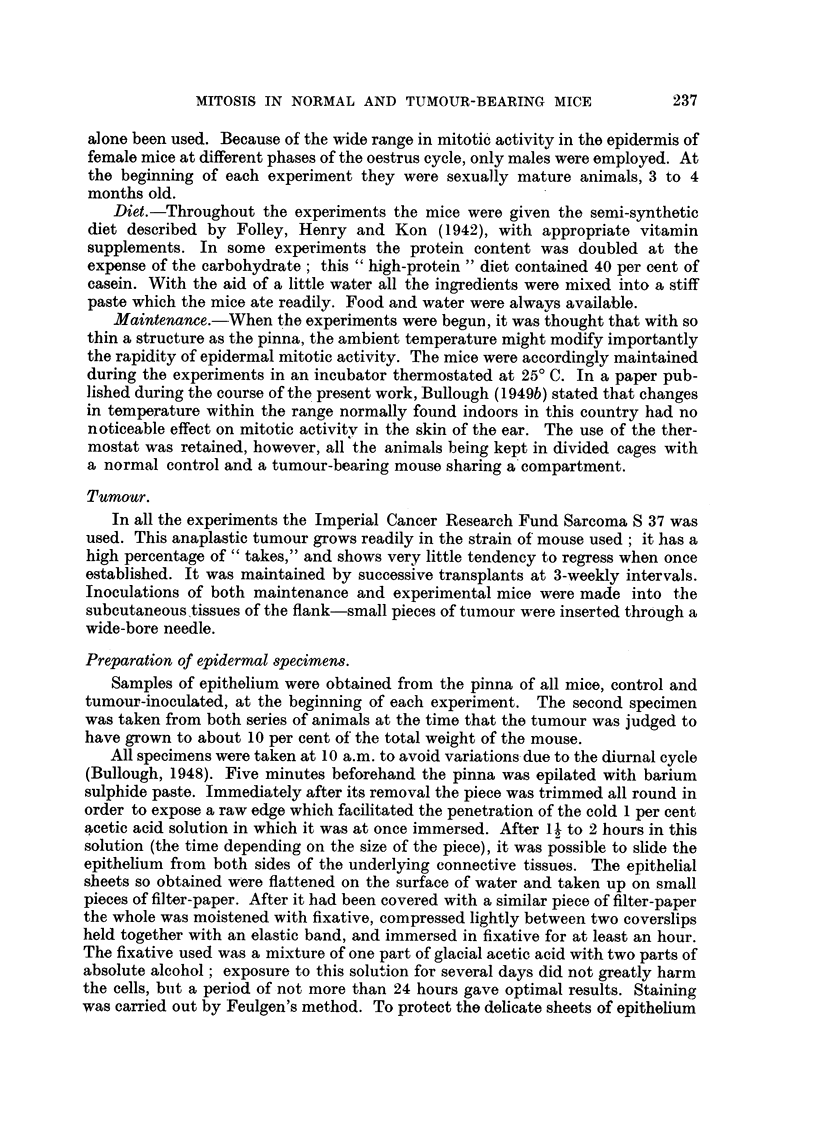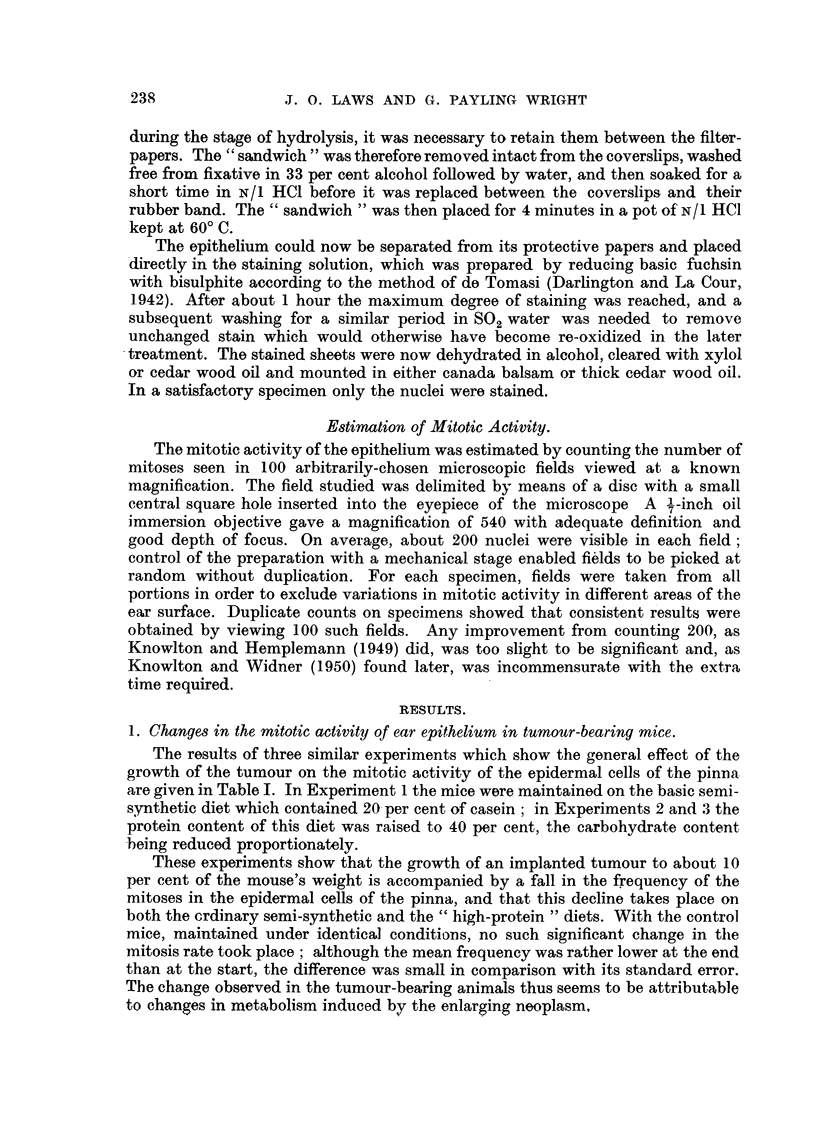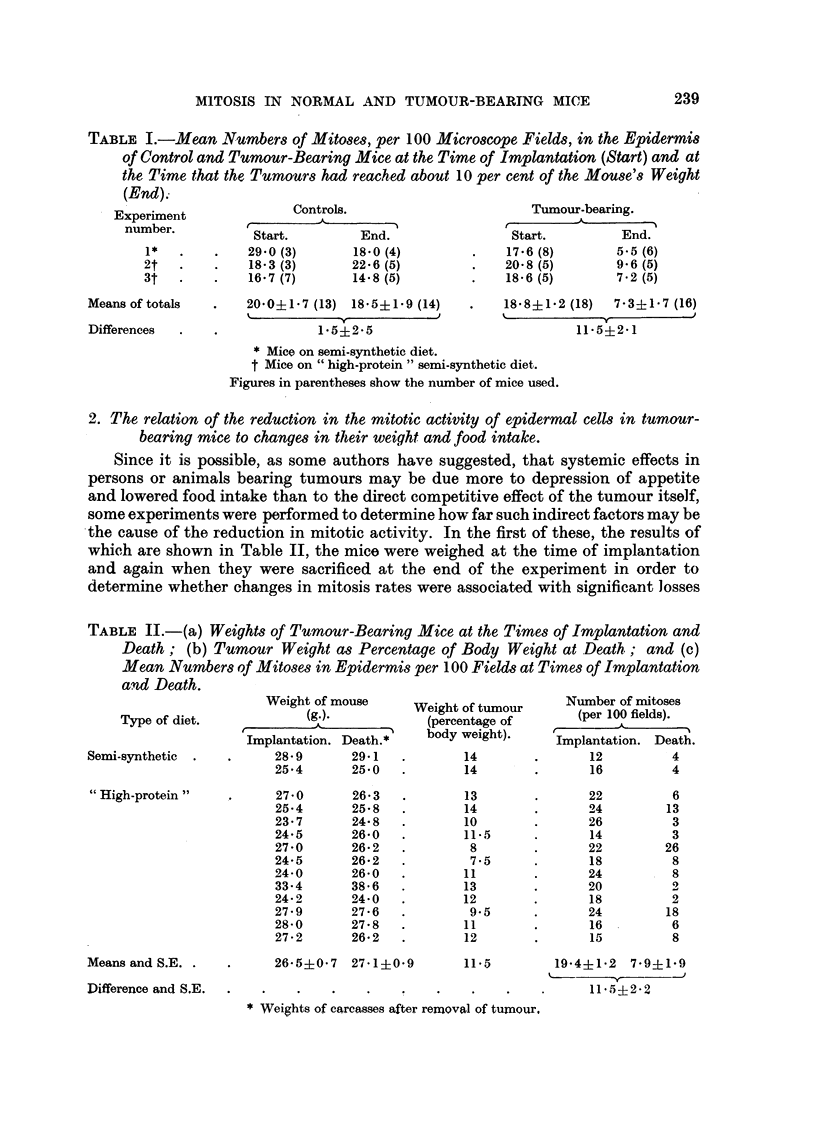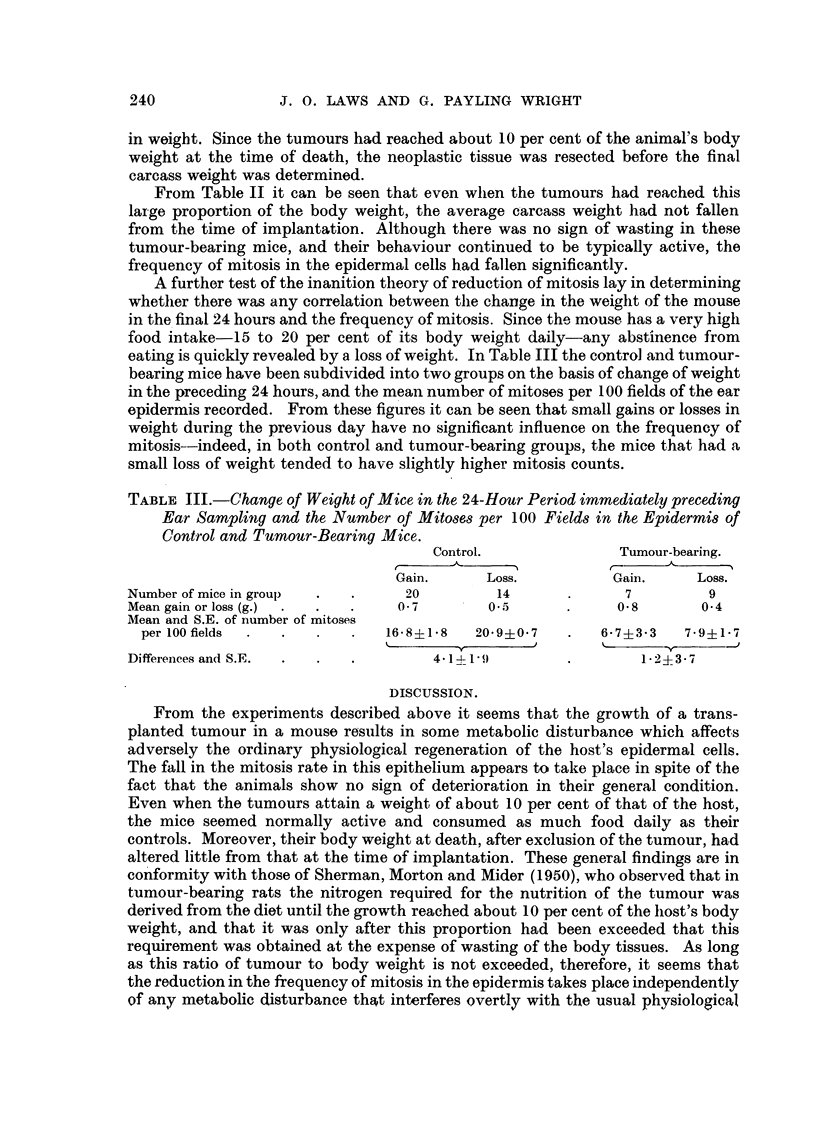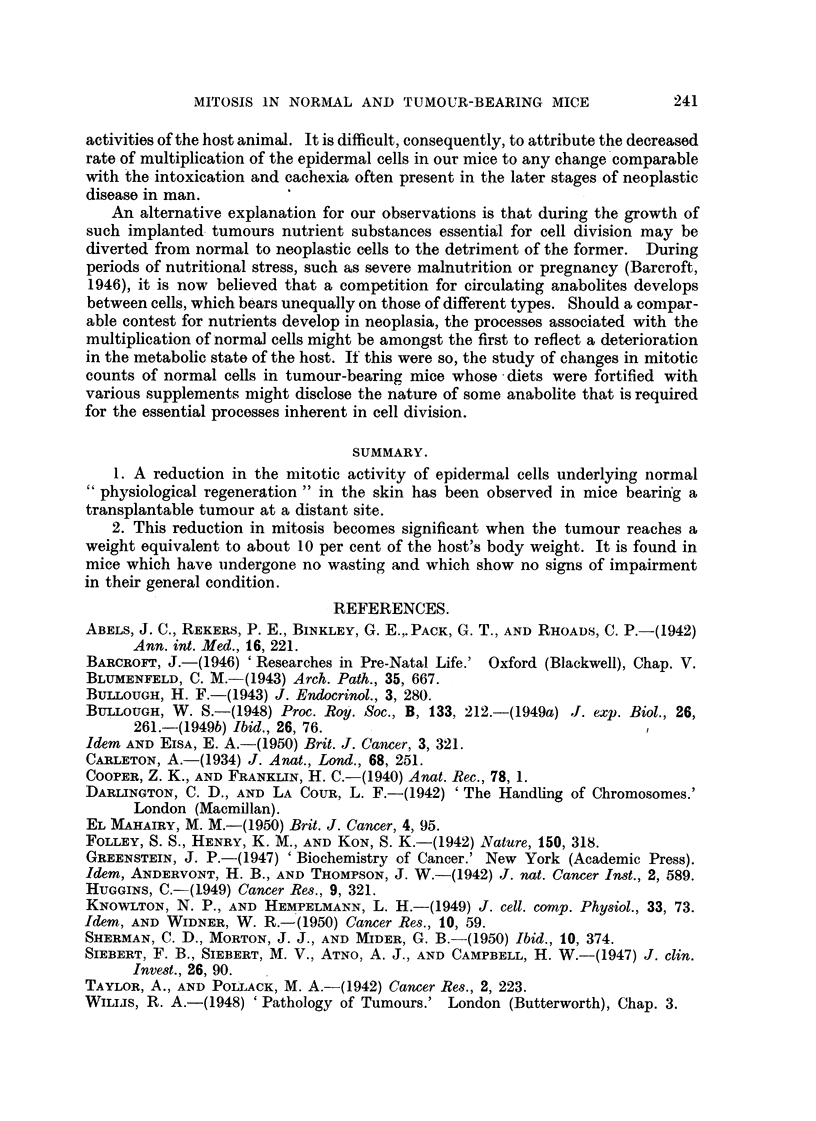# The Frequency of Mitosis in the Epidermis of Normal and Tumour-bearing Mice

**DOI:** 10.1038/bjc.1952.26

**Published:** 1952-09

**Authors:** J. O. Laws, G. Payling Wright


### THE FREQUENCY OF MITOSIS IN THE EPIDERMIS OF NORMAL AND TUMOUR-BEARING MICE.

J. O. LAWS AND G. PAYLING WRIGHT.

*From the Department of Pathology, Guy's Hospital Medical School.*

Received for publication August 8, 1952.

ALTHOUGH wasting and debility are common clinical findings in patients suffering from cancer, the nature of the relationship is still very obscure. Some authors (Willis, 1948) believe that the association is mainly indirect, and that the nutritional features result chiefly from anorexia, ulceration and infection, to which, with cancers of the alimentary tract especially, are added disordered digestion and absorption of food. Others consider that the connection may be more direct, perhaps through metabolic competition for circulating nutrients, and in recent years animal experiments have lent some support for this hypothesis. A reduction in plasma albumin concentration has been noted both in cancer patients and in tumour-bearing animals (Abels, Rekers, Binkley, Pack and Rhoads, 1942 ; Siebert, Siebert, Atno and Campbell, 1947 ; Huggins, 1949). An early fall in the blood haemoglobin level (Greenstein, Andervont and Thompson, 1942 ; Taylor and Pollack, 1942 ; El Mahairy, 1950) and marked reductions in tissue enzyme activity, e.g., in liver catalase, in organs far distant from the growth have been similarly recorded (Greenstein, 1947).

While there is no doubt that a growing cancer often exerts widespread effects on general metabolism, no study has yet been made of any process common to both normal and tumour cells in which any such metabolic competition might be expected to disclose itself. With this in mind, therefore, it seemed profitable to make a comparative study in normal and tumour-bearing animals of the occurrence of mitosis—a phase of the growth process which to the best of our present knowledge presents similar features in both types of cell. Accordingly, experiments have been undertaken to determine whether the presence of a growing transplanted tumour in a mouse is associated with any alteration in the frequency of mitotic cell division in normal host cells, as this is exemplified in the physiological regeneration of the epidermis.

### MATERIALS AND METHODS.

Owing to the known liability of the mitotic activity of epidermal cells to be affected by such factors as sex (Bullough, 1943), age (Bullough, 1949*a*), nutrition (Bullough and Eisa, 1950), time of day (Carleton, 1934 ; Cooper and Franklin, 1940 ; Blumenfeld, 1943 ; Bullough, 1948), as well as by such environmental conditions as temperature and humidity (Bullough, 1949*b*), precautions have been taken to minimize these recognized sources of variation.

### *Animals.*

To eliminate variations that might arise from the use of different strains, a sub-strain C of the Bagg albino mouse, maintained by brother-sister mating, has alone been used. Because of the wide range in mitotic activity in the epidermis of female mice at different phases of the oestrus cycle, only males were employed. At the beginning of each experiment they were sexually mature animals, 3 to 4 months old.

*Diet.*—Throughout the experiments the mice were given the semi-synthetic diet described by Folley, Henry and Kon (1942), with appropriate vitamin supplements. In some experiments the protein content was doubled at the expense of the carbohydrate ; this " high-protein " diet contained 40 per cent of casein. With the aid of a little water all the ingredients were mixed into a stiff paste which the mice ate readily. Food and water were always available.

*Maintenance.*—When the experiments were begun, it was thought that with so thin a structure as the pinna, the ambient temperature might modify importantly the rapidity of epidermal mitotic activity. The mice were accordingly maintained during the experiments in an incubator thermostated at 25° C. In a paper published during the course of the present work, Bullough (1949*b*) stated that changes in temperature within the range normally found indoors in this country had no noticeable effect on mitotic activity in the skin of the ear. The use of the thermostat was retained, however, all the animals being kept in divided cages with a normal control and a tumour-bearing mouse sharing a compartment.

*Tumour.*

In all the experiments the Imperial Cancer Research Fund Sarcoma S 37 was used. This anaplastic tumour grows readily in the strain of mouse used ; it has a high percentage of " takes," and shows very little tendency to regress when once established. It was maintained by successive transplants at 3-weekly intervals. Inoculations of both maintenance and experimental mice were made into the subcutaneous tissues of the flank—small pieces of tumour were inserted through a wide-bore needle.

*Preparation of epidermal specimens.*

Samples of epithelium were obtained from the pinna of all mice, control and tumour-inoculated, at the beginning of each experiment. The second specimen was taken from both series of animals at the time that the tumour was judged to have grown to about 10 per cent of the total weight of the mouse.

All specimens were taken at 10 a.m. to avoid variations due to the diurnal cycle (Bullough, 1948). Five minutes beforehand the pinna was epilated with barium sulphide paste. Immediately after its removal the piece was trimmed all round in order to expose a raw edge which facilitated the penetration of the cold 1 per cent acetic acid solution in which it was at once immersed. After 1½ to 2 hours in this solution (the time depending on the size of the piece), it was possible to slide the epithelium from both sides of the underlying connective tissues. The epithelial sheets so obtained were flattened on the surface of water and taken up on small pieces of filter-paper. After it had been covered with a similar piece of filter-paper the whole was moistened with fixative, compressed lightly between two coverslips held together with an elastic band, and immersed in fixative for at least an hour. The fixative used was a mixture of one part of glacial acetic acid with two parts of absolute alcohol ; exposure to this solution for several days did not greatly harm the cells, but a period of not more than 24 hours gave optimal results. Staining was carried out by Feulgen's method. To protect the delicate sheets of epithelium during the stage of hydrolysis, it was necessary to retain them between the filter-papers. The "sandwich" was therefore removed intact from the coverslips, washed free from fixative in 33 per cent alcohol followed by water, and then soaked for a short time in N/1 HCl before it was replaced between the coverslips and their rubber band. The " sandwich " was then placed for 4 minutes in a pot of N/1 HCl kept at 60° C.

The epithelium could now be separated from its protective papers and placed directly in the staining solution, which was prepared by reducing basic fuchsin with bisulphite according to the method of de Tomasi (Darlington and La Cour, 1942). After about 1 hour the maximum degree of staining was reached, and a subsequent washing for a similar period in $SO_2$ water was needed to remove unchanged stain which would otherwise have become re-oxidized in the later treatment. The stained sheets were now dehydrated in alcohol, cleared with xylol or cedar wood oil and mounted in either canada balsam or thick cedar wood oil. In a satisfactory specimen only the nuclei were stained.

*Estimation of Mitotic Activity.*

The mitotic activity of the epithelium was estimated by counting the number of mitoses seen in 100 arbitrarily-chosen microscopic fields viewed at a known magnification. The field studied was delimited by means of a disc with a small central square hole inserted into the eyepiece of the microscope A $\frac{1}{7}$-inch oil immersion objective gave a magnification of 540 with adequate definition and good depth of focus. On average, about 200 nuclei were visible in each field ; control of the preparation with a mechanical stage enabled fields to be picked at random without duplication. For each specimen, fields were taken from all portions in order to exclude variations in mitotic activity in different areas of the ear surface. Duplicate counts on specimens showed that consistent results were obtained by viewing 100 such fields. Any improvement from counting 200, as Knowlton and Hemplemann (1949) did, was too slight to be significant and, as Knowlton and Widner (1950) found later, was incommensurate with the extra time required.

RESULTS.

1. *Changes in the mitotic activity of ear epithelium in tumour-bearing mice.*

The results of three similar experiments which show the general effect of the growth of the tumour on the mitotic activity of the epidermal cells of the pinna are given in Table I. In Experiment 1 the mice were maintained on the basic semi-synthetic diet which contained 20 per cent of casein ; in Experiments 2 and 3 the protein content of this diet was raised to 40 per cent, the carbohydrate content being reduced proportionately.

These experiments show that the growth of an implanted tumour to about 10 per cent of the mouse's weight is accompanied by a fall in the frequency of the mitoses in the epidermal cells of the pinna, and that this decline takes place on both the ordinary semi-synthetic and the " high-protein " diets. With the control mice, maintained under identical conditions, no such significant change in the mitosis rate took place ; although the mean frequency was rather lower at the end than at the start, the difference was small in comparison with its standard error. The change observed in the tumour-bearing animals thus seems to be attributable to changes in metabolism induced by the enlarging neoplasm.

TABLE I.—*Mean Numbers of Mitoses, per 100 Microscope Fields, in the Epidermis of Control and Tumour-Bearing Mice at the Time of Implantation (Start) and at the Time that the Tumours had reached about 10 per cent of the Mouse's Weight (End).*

| Experiment number. | Controls. | | Tumour-bearing. | |
|---|---|---|---|---|
| | Start. | End. | Start. | End. |
| 1* | 29·0 (3) | 18·0 (4) | 17·6 (8) | 5·5 (6) |
| 2† | 18·3 (3) | 22·6 (5) | 20·8 (5) | 9·6 (5) |
| 3† | 16·7 (7) | 14·8 (5) | 18·6 (5) | 7·2 (5) |
| Means of totals | 20·0±1·7 (13) | 18·5±1·9 (14) | 18·8±1·2 (18) | 7·3±1·7 (16) |
| Differences | 1·5±2·5 | | 11·5±2·1 | |

* Mice on semi-synthetic diet.
† Mice on " high-protein " semi-synthetic diet.
Figures in parentheses show the number of mice used.

2. *The relation of the reduction in the mitotic activity of epidermal cells in tumour-bearing mice to changes in their weight and food intake.*

Since it is possible, as some authors have suggested, that systemic effects in persons or animals bearing tumours may be due more to depression of appetite and lowered food intake than to the direct competitive effect of the tumour itself, some experiments were performed to determine how far such indirect factors may be the cause of the reduction in mitotic activity. In the first of these, the results of which are shown in Table II, the mice were weighed at the time of implantation and again when they were sacrificed at the end of the experiment in order to determine whether changes in mitosis rates were associated with significant losses

TABLE II.—(a) *Weights of Tumour-Bearing Mice at the Times of Implantation and Death* ; (b) *Tumour Weight as Percentage of Body Weight at Death* ; *and* (c) *Mean Numbers of Mitoses in Epidermis per 100 Fields at Times of Implantation and Death.*

| Type of diet. | Weight of mouse (g.). | | Weight of tumour (percentage of body weight). | Number of mitoses (per 100 fields). | |
|---|---|---|---|---|---|
| | Implantation. | Death.* | | Implantation. | Death. |
| Semi-synthetic | 28·9 | 29·1 | 14 | 12 | 4 |
| | 25·4 | 25·0 | 14 | 16 | 4 |
| " High-protein " | 27·0 | 26·3 | 13 | 22 | 6 |
| | 25·4 | 25·8 | 14 | 24 | 13 |
| | 23·7 | 24·8 | 10 | 26 | 3 |
| | 24·5 | 26·0 | 11·5 | 14 | 3 |
| | 27·0 | 26·2 | 8 | 22 | 26 |
| | 24·5 | 26·2 | 7·5 | 18 | 8 |
| | 24·0 | 26·0 | 11 | 24 | 8 |
| | 33·4 | 38·6 | 13 | 20 | 2 |
| | 24·2 | 24·0 | 12 | 18 | 2 |
| | 27·9 | 27·6 | 9·5 | 24 | 18 |
| | 28·0 | 27·8 | 11 | 16 | 6 |
| | 27·2 | 26·2 | 12 | 15 | 8 |
| Means and S.E. | 26·5±0·7 | 27·1±0·9 | 11·5 | 19·4±1·2 | 7·9±1·9 |
| Difference and S.E. | | | | 11·5±2·2 | |

* Weights of carcasses after removal of tumour.

in weight. Since the tumours had reached about 10 per cent of the animal's body weight at the time of death, the neoplastic tissue was resected before the final carcass weight was determined.

From Table II it can be seen that even when the tumours had reached this large proportion of the body weight, the average carcass weight had not fallen from the time of implantation. Although there was no sign of wasting in these tumour-bearing mice, and their behaviour continued to be typically active, the frequency of mitosis in the epidermal cells had fallen significantly.

A further test of the inanition theory of reduction of mitosis lay in determining whether there was any correlation between the change in the weight of the mouse in the final 24 hours and the frequency of mitosis. Since the mouse has a very high food intake—15 to 20 per cent of its body weight daily—any abstinence from eating is quickly revealed by a loss of weight. In Table III the control and tumour-bearing mice have been subdivided into two groups on the basis of change of weight in the preceding 24 hours, and the mean number of mitoses per 100 fields of the ear epidermis recorded. From these figures it can be seen that small gains or losses in weight during the previous day have no significant influence on the frequency of mitosis—indeed, in both control and tumour-bearing groups, the mice that had a small loss of weight tended to have slightly higher mitosis counts.

TABLE III.—*Change of Weight of Mice in the 24-Hour Period immediately preceding Ear Sampling and the Number of Mitoses per 100 Fields in the Epidermis of Control and Tumour-Bearing Mice.*

| | Control. | | Tumour-bearing. | |
|---|---|---|---|---|
| | Gain. | Loss. | Gain. | Loss. |
| Number of mice in group | 20 | 14 | 7 | 9 |
| Mean gain or loss (g.) | 0·7 | 0·5 | 0·8 | 0·4 |
| Mean and S.E. of number of mitoses per 100 fields | 16·8±1·8 | 20·9±0·7 | 6·7±3·3 | 7·9±1·7 |
| Differences and S.E. | 4·1±1·9 | | 1·2±3·7 | |

DISCUSSION.

From the experiments described above it seems that the growth of a transplanted tumour in a mouse results in some metabolic disturbance which affects adversely the ordinary physiological regeneration of the host's epidermal cells. The fall in the mitosis rate in this epithelium appears to take place in spite of the fact that the animals show no sign of deterioration in their general condition. Even when the tumours attain a weight of about 10 per cent of that of the host, the mice seemed normally active and consumed as much food daily as their controls. Moreover, their body weight at death, after exclusion of the tumour, had altered little from that at the time of implantation. These general findings are in conformity with those of Sherman, Morton and Mider (1950), who observed that in tumour-bearing rats the nitrogen required for the nutrition of the tumour was derived from the diet until the growth reached about 10 per cent of the host's body weight, and that it was only after this proportion had been exceeded that this requirement was obtained at the expense of wasting of the body tissues. As long as this ratio of tumour to body weight is not exceeded, therefore, it seems that the reduction in the frequency of mitosis in the epidermis takes place independently of any metabolic disturbance that interferes overtly with the usual physiological activities of the host animal. It is difficult, consequently, to attribute the decreased rate of multiplication of the epidermal cells in our mice to any change comparable with the intoxication and cachexia often present in the later stages of neoplastic disease in man.

An alternative explanation for our observations is that during the growth of such implanted tumours nutrient substances essential for cell division may be diverted from normal to neoplastic cells to the detriment of the former. During periods of nutritional stress, such as severe malnutrition or pregnancy (Barcroft, 1946), it is now believed that a competition for circulating anabolites develops between cells, which bears unequally on those of different types. Should a comparable contest for nutrients develop in neoplasia, the processes associated with the multiplication of normal cells might be amongst the first to reflect a deterioration in the metabolic state of the host. If this were so, the study of changes in mitotic counts of normal cells in tumour-bearing mice whose diets were fortified with various supplements might disclose the nature of some anabolite that is required for the essential processes inherent in cell division.

SUMMARY.

1. A reduction in the mitotic activity of epidermal cells underlying normal "physiological regeneration" in the skin has been observed in mice bearing a transplantable tumour at a distant site.

2. This reduction in mitosis becomes significant when the tumour reaches a weight equivalent to about 10 per cent of the host's body weight. It is found in mice which have undergone no wasting and which show no signs of impairment in their general condition.

### REFERENCES.

Abels, J. C., Rekers, P. E., Binkley, G. E., Pack, G. T., and Rhoads, C. P.—(1942) *Ann. int. Med.*, **16**, 221.
Barcroft, J.—(1946) 'Researches in Pre-Natal Life.' Oxford (Blackwell), Chap. V.
Blumenfeld, C. M.—(1943) *Arch. Path.*, **35**, 667.
Bullough, H. F.—(1943) *J. Endocrinol.*, **3**, 280.
Bullough, W. S.—(1948) *Proc. Roy. Soc.*, **B**, **133**, 212.—(1949*a*) *J. exp. Biol.*, **26**, 261.—(1949*b*) *Ibid.*, **26**, 76.
*Idem* and Eisa, E. A.—(1950) *Brit. J. Cancer*, **3**, 321.
Carleton, A.—(1934) *J. Anat., Lond.*, **68**, 251.
Cooper, Z. K., and Franklin, H. C.—(1940) *Anat. Rec.*, **78**, 1.
Darlington, C. D., and La Cour, L. F.—(1942) 'The Handling of Chromosomes.' London (Macmillan).
El Mahairy, M. M.—(1950) *Brit. J. Cancer*, **4**, 95.
Folley, S. S., Henry, K. M., and Kon, S. K.—(1942) *Nature*, **150**, 318.
Greenstein, J. P.—(1947) 'Biochemistry of Cancer.' New York (Academic Press).
*Idem*, Andervont, H. B., and Thompson, J. W.—(1942) *J. nat. Cancer Inst.*, **2**, 589.
Huggins, C.—(1949) *Cancer Res.*, **9**, 321.
Knowlton, N. P., and Hempelmann, L. H.—(1949) *J. cell. comp. Physiol.*, **33**, 73.
*Idem*, and Widner, W. R.—(1950) *Cancer Res.*, **10**, 59.
Sherman, C. D., Morton, J. J., and Mider, G. B.—(1950) *Ibid.*, **10**, 374.
Siebert, F. B., Siebert, M. V., Atno, A. J., and Campbell, H. W.—(1947) *J. clin. Invest.*, **26**, 90.
Taylor, A., and Pollack, M. A.—(1942) *Cancer Res.*, **2**, 223.
Willis, R. A.—(1948) 'Pathology of Tumours.' London (Butterworth), Chap. 3.